# Tanshinone IIA Downregulates Lipogenic Gene Expression and Attenuates Lipid Accumulation through the Modulation of LXRα/SREBP1 Pathway in HepG2 Cells

**DOI:** 10.3390/biomedicines9030326

**Published:** 2021-03-23

**Authors:** Wan-Yun Gao, Pei-Yi Chen, Hao-Jen Hsu, Ching-Yen Lin, Ming-Jiuan Wu, Jui-Hung Yen

**Affiliations:** 1 Institute of Medical Sciences, Tzu Chi University, Hualien 970, Taiwan; 102712131@gms.tcu.edu.tw; 2 Center of Medical Genetics, Hualien Tzu Chi Hospital, Buddhist Tzu Chi Medical Foundation, Hualien 970, Taiwan; pyc571@gmail.com; 3 Department of Molecular Biology and Human Genetics, Tzu Chi University, Hualien 970, Taiwan; jouyuan22@gmail.com; 4 Department of Life Science, Tzu Chi University, Hualien 970, Taiwan; hjhsu32@mail.tcu.edu.tw; 5 Department of Biotechnology, Chia Nan University of Pharmacy and Science, Tainan 717, Taiwan; mingjiuanwu@gmail.com

**Keywords:** MAFLD, tanshinone IIA, phytochemical, lipogenesis, lipid accumulation, LXRα

## Abstract

Abnormal and excessive accumulation of lipid droplets within hepatic cells is the main feature of steatosis and nonalcoholic fatty liver disease (NAFLD) or metabolic-associated fatty liver disease (MAFLD). Dysregulation of lipogenesis contributes to hepatic steatosis and plays an essential role in the pathological progress of MAFLD. Tanshinone IIA is a bioactive phytochemical isolated from *Salvia miltiorrhiza* Bunge and exhibits anti-inflammatory, antiatherosclerotic and antihyperlipidemic effects. In this study, we aimed to investigate the lipid-lowering effects of tanshinone IIA on the regulation of lipogenesis, lipid accumulation, and the underlying mechanisms in hepatic cells. We demonstrated that tanshinone IIA can significantly inhibit the gene expression involved in de novo lipogenesis including FASN, ACC1, and SCD1, in HepG2 and Huh 7 cells. Tanshinone IIA could increase phosphorylation of ACC1 protein in HepG2 cells. We further demonstrated that tanshinone IIA also could suppress the fatty-acid-induced lipogenesis and TG accumulation in HepG2 cells. Furthermore, tanshinone IIA markedly downregulated the mRNA and protein expression of SREBP1, an essential transcription factor regulating lipogenesis in hepatic cells. Moreover, we found that tanshinone IIA attenuated liver X receptor α (LXRα)-mediated lipogenic gene expression and lipid droplet accumulation, but did not change the levels of LXRα mRNA or protein in HepG2 cells. The molecular docking data predicted tanshinone IIA binding to the ligand-binding domain of LXRα, which may result in the attenuation of LXRα-induced transcriptional activation. Our findings support the supposition that tanshinone IIA possesses a lipid-modulating effect that suppresses lipogenesis and attenuates lipid accumulation by modulating the LXRα/SREBP1 pathway in hepatic cells. Tanshinone IIA can be potentially used as a supplement or drug for the prevention or treatment of MAFLD.

## 1. Introduction

The liver is a major organ in the regulation of lipid metabolism and is critical for lipid acquisition, storage, export, and utilization as energy upon the oxidation of fatty acids [1]. The disruption of hepatic lipid homeostasis, such as that realized when balance between lipid acquisition through fatty-acid uptake and de novo lipogenesis is disrupted, and lipid deposition may lead to fat retention within the liver and the consequential development of nonalcoholic fatty liver disease (NAFLD), renamed as metabolic-associated fatty liver disease (MAFLD) [2,3,4,5]. MAFLD is recognized as the most common form of chronic liver disease worldwide, and it can cause deleterious clinical problems, including end-stage liver diseases and cancer, in some cases. MAFLD is frequently associated with insulin resistance, type 2 diabetes mellitus, hyperlipidemia, and obesity [6,7] The main feature of MAFLD pathogenesis is the accumulation of lipids in the liver. MAFLD manifests in liver conditions ranging from simple triglyceride (TG) and cholesterol accumulation to steatosis. Abnormal and excessive lipid accumulation in the liver has the potential to induce inflammatory injury in hepatocytes and lead to nonalcoholic steatohepatitis (NASH). NASH can progress to advanced fibrosis, cirrhosis, and hepatocellular carcinoma, and result in increases in liver-related mortality [8,9]. To date, no approved agents are available for the prevention and treatment of MAFLD. Thus, the development of therapeutic compounds for MAFLD is currently being actively pursued [10,11].

The lipid biosynthesis process plays the most important role in the regulation of fat accumulation in the liver. In hepatic cells, the first step of de novo fatty-acid synthesis is the conversion of acetyl-CoA to malonyl-CoA by acetyl-CoA carboxylase 1 (ACC1), then malonyl-CoA is converted to palmitate through a reaction catalyzed by a multifunctional fatty-acid synthase (FASN). Palmitate may further undergo elongation catalyzed by long-chain elongase, desaturation catalyzed by stearoyl-CoA desaturase 1 (SCD1), and esterification for triglyceride production or exportation by very low-density lipoprotein (VLDL) particles [12,13,14,15]. It has been demonstrated that increases in de novo lipogenesis in the liver may cause hypertriglyceridemia and play an essential role in MAFLD pathogenesis [16]. MAFLD patients were reported to have increased abnormal de novo lipogenesis even in fasting condition. The clinical data also showed that failure to regulate the process of de novo lipogenesis may cause hepatic lipid accumulation in obese patients with MAFLD [17]. These findings indicate that modulation of de novo lipogenesis in the liver can provide useful strategies to prevent hepatic steatosis and the development of MAFLD.

The transcriptional regulation of de novo lipogenesis is predominantly regulated by sterol regulatory element binding protein 1 (SREBP1), which binds to the sterol regulatory element (SRE) and upregulates the gene expression of FASN, ACC1, and SCD1, thereby increasing TG production and accumulation in hepatic cells [18]. It is known that SREBP-1c expression is stimulated by the transcription factor liver X receptor α (LXRα), a ligand-activated nuclear hormone receptor [19]. LXRα and retinoid X receptor (RXR) can form a heterodimer that binds to LXR-responsive elements (LXREs) in the promoter of target genes. LXRα binding to ligands causes the release of repressors and the recruitment of coactivators that interact with the LXRα/RXR heterodimer for the induction of target gene transcription [20,21]. It has been demonstrated that distinct LXRα ligands, such as synthetic nonsteroidal ligands or endogenous oxysterols, induce LXRα activation and promote de novo lipogenesis in the liver, which may lead to elevated levels of plasma and hepatic lipid [22,23,24,25]. Modulation of the LXRα/SREBP1-dependent lipogenesis pathway may be beneficial for reducing hepatic lipid accumulation and treating MAFLD.

Tanshinone IIA is a main lipophilic diterpenoids isolated from *Salvia miltiorrhiza* Bunge, also known as Danshen, with antioxidant, anti-inflammation, antiatherosclerosis, and anticancer properties that can be used in the prevention or treatment of hepatic fibrosis, neurodegeneration, and cardiovascular diseases [26,27]. Recently, several in vitro and in vivo studies have suggested that tanshinone IIA can modulate lipid homeostasis. Tanshinone IIA can attenuate oxidized-LDL (oxLDL) uptake by macrophage and inhibit foam-cell formation [28,29]. Tanshinone IIA can also increase LDLR levels and LDL uptake by cells through the downregulation of PCSK9 expression in HepG2 cells [30] Tanshinone IIA was reported to attenuate atherosclerotic lesions in hyperlipidemic and hypercholesterolemic animal models [31,32] Tanshinone IIA regulated the SREBP2/PCSK9 pathway, increased the levels of HDL, and decreased lipid deposition in the livers of hyperlipidemic rats [33,34]. In rats fed a high-fat diet, tanshinone IIA treatment reduced fat accumulation and partly ameliorated oxidative stress, inflammation, and apoptosis in the livers [35].

These recent findings suggest that tanshinone IIA can modulate hepatic lipid metabolism and homeostasis; however, the underlying mechanisms by which tanshinone IIA regulates hepatic lipid homeostasis remain unclear. In this study, we aimed to investigate the lipid-modulating effect and underlying mechanisms of tanshinone IIA, particularly focusing on its effects on lipogenic gene expression and lipid accumulation in hepatic cells.

## 2. Materials and Methods

### 2.1. Chemicals

Tanshinone IIA, 3-(4,5-dimethylthiazol-2-yl)-2,5-diphenyltetrazolium bromide (MTT), dimethyl sulfoxide (DMSO), palmitic acid, oleic acid, insulin–transferrin–selenium (ITS), T0901317, 24(S),25-epoxycholesterol, and other chemicals were purchased from Sigma-Aldrich Co. (St. Louis, MO, USA) unless otherwise indicated. DMEM, fetal bovine serum (FBS) and nonessential amino acids (NEAAs) were purchased from Thermo Fisher Scientific, Inc. (Rockford, IL, USA).

### 2.2. Cell Culture and Compound Treatment

HepG2 and Huh7 cells were cultured in growth medium composed of DMEM containing 10% FBS and 1x nonessential amino acid (NEAA) solution in a 5% CO_2_ incubator at 37 °C. The high-fat medium formula was described in previous literature and slightly modified [36,37]. For the high-fat medium preparation, stock solutions of palmitic acid (0.25 mM) and oleic acid (1 mM) were prepared and precomplexed with 10% FBS in DMEM medium, respectively, for dilution of the desired final concentration. The high-fat medium contained DMEM, 10% FBS, 1xNEAA, palmitic acid (0.125 mM), oleic acid (0.5 mM), and insulin–transferrin–selenium (ITS). For compound treatment for 24 h in the high-fat medium condition, cells were treated with vehicle (0.1% DMSO) or tanshinone IIA (1–10 μM) in growth medium, and after 20 h, the medium was replaced with high-fat medium containing the indicated compounds (vehicle or tanshinone IIA) and incubated for an additional 4 h. For LXRα ligand treatment, cells were pretreated with vehicle or tanshinone IIA (10 μM) for 1 h, followed by incubation with T0901317 (1 μM) or 24(S),25-epoxycholesterol (40 μM) for 24 h and harvested for further studies.

### 2.3. Analysis of Cell Viability by MTT Assay

The viability of cells was determined by MTT assay as described previously [38]. Cells were treated with vehicle or tanshinone IIA (1–20 μM) for 24 h. These cells were incubated with the MTT reagent (1 mg/mL) at 37 °C for 3 h. The medium was removed, and the cells were washed with PBS. Purple crystals were dissolved in DMSO, and cell viability was determined by the absorbance measured at 550 nm using a microplate reader.

### 2.4. Analysis of Cell Cytotoxicity by LDH Release Assay

The cytotoxicity of cells was determined by LDH assay as described previously [39] Cells were treated with vehicle or tanshinone IIA (1–20 μM) for 24 h. Supernatants were harvested and LDH activity was measured using an LDH Cytotoxicity Assay Kit II (Abcam, Cambridge, MA, USA) according to the manufacturer’s protocol. The absorbance of samples was determined at 450 nm using a microplate reader.

### 2.5. Reverse-Transcription Quantitative Polymerase Chain Reaction (RT-qPCR) Analysis

The RT-qPCR analysis was carried out as described previously [40] Cells were treated with vehicle or tanshinone IIA (5 and 10 μM) for 24 h, and cellular RNA was extracted using a FavorPrep^TM^ blood/cultured cell total RNA purification mini kit (FAVORGEN Biotech, Ping-Tung, Taiwan). Reverse transcription was carried out with a high-capacity cDNA reverse transcription kit (Thermo Fisher Scientific, MA, USA). Quantitative real-time PCR was performed in a mixture containing cDNA, human-specific primers (Table S1), and Maxima SYBR Green/ROX qPCR Master Mix (Thermo Fisher Scientific) on a Roche LightCycler^®^-480 real-time PCR system. The ∆∆C_t_ method was used to measure the relative differences in mRNA expression between experimental groups and normalize with the mRNA expression of GAPDH in the same samples.

### 2.6. Western Blot Analysis

Western blot analysis was carried out as described previously [41]. Cells were treated with vehicle or tanshinone IIA (5 and 10 μM) for 24 h. For preparation of total cellular proteins, the cells were harvested with RIPA buffer (Thermo Fisher Scientific). For nuclear-extract preparation, the cells were harvested using NE-PER nuclear and cytoplasmic extraction reagent (Thermo Fisher Scientific). The samples were separated by 10% SDS-PAGE and transferred to PVDF membranes (PerkinElmer, Boston, MA, USA). The blots were incubated with the following primary antibodies at 4 °C for 24 h: anti-FASN (A6273) (1:2000) (ABclonal, Woburn, MA, USA), anti-ACC1 (#3676) (1:1000) and anti-phospho-ACC1 (Ser79) (#3661) (1:1000) (Cell Signaling Technology, Danvers, MA), anti-SCD1 (ab39969) (1:1000) and anti-LXRα (ab41902) (1:1000) (Abcam), anti-HDAC2 (GTX112957) (1:3000) (GeneTex, Irvine, CA, USA), anti-SREBP1 (sc-13551) (1:200) (Santa Cruz Biotechnology, Santa Cruz, CA, USA) and anti-actin (MAB1501) (1:30000) (Thermo Fisher Scientific). The blots were incubated with the appropriate HRP-conjugated secondary antibodies, and the amount of each protein was measured by Amersham ECL^TM^ prime Western blotting detection reagent. The chemiluminescent signal was visualized using Amersham Hyperfilm^TM^ ECL film (GE Healthcare, Buckinghamshire, UK).

### 2.7. Staining of Neutral Lipid Droplets

Lipid droplets were stained by a specifically lipophilic fluorescent dye as described previously [42,43]. HepG2 cells were seeded in poly-L-lysine-coated coverslips and treated with vehicle or tanshinone IIA in growth medium or high-fat medium as described above. The cells on coverslips were fixed in cold 4% paraformaldehyde at room temperature for 20 min followed by incubation with 0.5 μM BODIPY493/503 fluorescent dye (Thermo Fisher Scientific) for 15 min. The coverslips were washed three times with PBS and mounted using VECTASHIELD HardSet™ mounting medium with 4’,6-diamidino-2-phenylindole (DAPI) (Vector Laboratories, Burlingame, CA, USA). The imaged were observed and photographed with a ZEISS LSM 900 laser confocal microscope (ZEISS Microscopy, Jena, Germany). The mean fluorescent intensities of the lipid droplets per cell were analyzed and quantified in at least 200 cells in 25–30 randomly selected fields (63x) from independent replicates using ZEISS ZEN lite software (ZEN 2.3) (ZEISS Microscopy).

### 2.8. Analysis of Hepatic Triglyceride (TG)

The cells were treated with vehicle or tanshinone IIA, and cell lysates were prepared by using sonication. The triglyceride (TG) was extracted from cell lysates as described previously [44]. The amounts of TG were measured with a Triglyceride Colorimetric Assay Kit (Cayman Chemical, Ann Arbor, MI, USA) according to the manufacturer’s protocol. The TG content was measured as the absorbance at 530 nm using a microplate reader.

### 2.9. Molecular Docking of Compounds to Binding Domain of LXRα Protein

The molecular docking of compounds to nuclear receptor LXRα was performed using Molecular Operating Environment software (MOE2019.01). The DOCK module with “induced fit” refinement to enhance the accuracy of the MOE2019.01 software program was used to predict the preferable binding sites between LXRα and the respective compounds (tanshinone IIA, T0901317, and 24 (S),25-epoxycholesterol). The compounds were manually built with the MOE software package for docking with the LXRα binding domain (PDB code: 2ACL, LXRα with compound SB313987). Water molecules in the crystal structure were removed, missing short loops were added with the MOE software, and the energy was minimized before molecular docking. The score was based on GBVI/WSA ΔG, a force-field-based scoring function that estimates the binding free energy of a ligand to a receptor. The preferable binding sites for each compound were determined based on the lowest binding free energy, which was the lowest S value in the scoring function.

### 2.10. Statistical Analysis

At least three independent experiments were performed, and each experiment was repeated three times. The data are expressed as the means ± SD. The data were analyzed using Student’s *t*-test (for comparisons of data with two groups) and one-way ANOVA with Tukey’s test for post hoc analysis (for comparisons of data with multiple groups), and a *p*-value of <0.05 was considered statistically significant.

## 3. Results

### 3.1. Effects of Tanshinone IIA on the Viability of Hepatic Cell Lines

**Figure 1:**
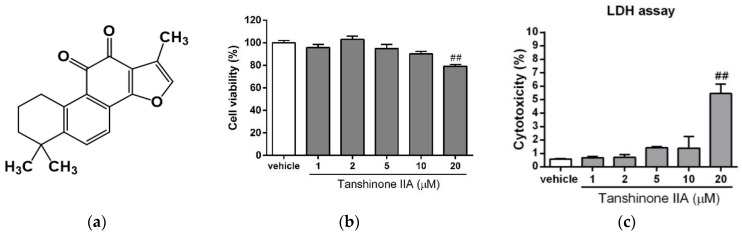
Effects of tanshinone IIA on HepG2 cell viability. (**a**) The chemical structure of tanshinone IIA. HepG2 cells were treated with vehicle (0.1% DMSO) or tanshinone IIA (1, 2, 5, 10, and 20 μM) for 24 h. (**b**) Cell viability was measured using an MTT assay. (**c**) Cytotoxicity was measured using LDH release assay. The data represent the mean ± SD of three independent experiments. ## *p* < 0.01 indicates significant differences compared to the vehicle-treated cells.

To investigate the cytotoxic effect of tanshinone IIA (Figure 1a) on hepatic cells, HepG2 cells were treated with vehicle (0.1% DMSO) or tanshinone IIA (1, 2, 5, 10, and 20 μM) for 24 h. Cell cytotoxicity induced by tanshinone IIA was determined using an MTT assay and validated by an LDH release assay. The data showed that vehicle (0.1% DMSO) and tanshinone IIA (1–10 μM) induced no cytotoxicity in the HepG2 cells (Figure 1b,c). However, higher concentration of tanshinone IIA (20 μM) slightly reduced the cell viability as compared with vehicle-treated group. Similar results for cell viability also were found in the tanshinone IIA-treated Huh 7 hepatic cell line (Figure S1). Therefore, doses of 5 and 10 μM of tanshinone IIA were used in the subsequent experiments.

### 3.2. Effects of Tanshinone IIA on FASN, ACC1, and SCD1 Expression in Hepatic Cell Lines

**Figure 2:**
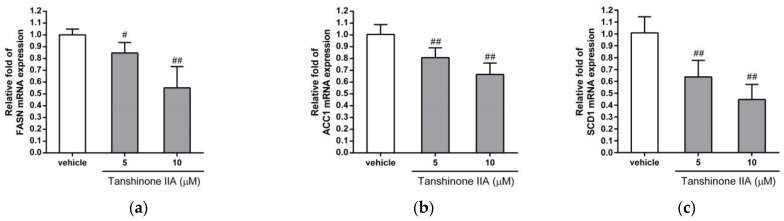
Effects of tanshinone IIA on FASN, ACC1, and SCD1 mRNA expression in HepG2 cells. HepG2 cells were treated with vehicle (0.1% DMSO) or tanshinone IIA (5 and 10 μM) for 24 h. The mRNA expression of (**a**) FASN, (**b**) ACC1, and (**c**) SCD1 was measured by RT-qPCR analysis. The data represent the mean ± SD of three independent experiments. # *p* < 0.05 and ## *p* < 0.01 indicate significant differences compared to the vehicle-treated cells.

De novo lipogenesis plays a critical role in regulating the amount of lipid accumulation in hepatic cells. To investigate whether tanshinone IIA can regulate the expression of endogenous de novo lipogenesis-related genes, we examined the effect of tanshinone IIA treatment on the mRNA expression of fatty-acid synthase (FASN), acetyl-CoA carboxylase 1 (ACC1), and stearoyl-CoA desaturase 1 (SCD1) in HepG2 cells. Tanshinone IIA (5 and 10 μM) significantly reduced the mRNA expression of FASN, ACC1, and SCD1 by 0.85 ± 0.09- and 0.55 ± 0.18-fold; 0.81 ± 0.08- and 0.67 ± 0.10-fold; and 0.64 ± 0.14- and 0.45 ± 0.13-fold in HepG2 cells, respectively, when compared to the vehicle-treated cells (1.00 ± 0.05, 1.00 ± 0.08, and 1.01 ± 0.14) (*p* < 0.05 and *p* < 0.01, respectively) (Figure 2a–c). Similar results showed that tanshinone IIA could significantly inhibit the mRNA expression of FASN, ACC1, and SCD1 genes in Huh 7 cells (Figure S2a–c).

**Figure 3:**
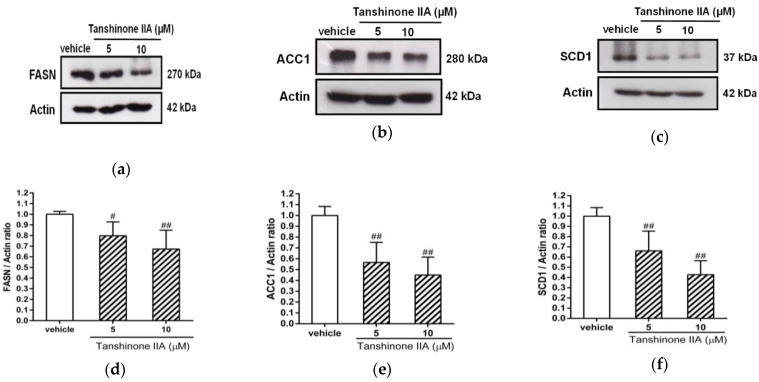
Effects of tanshinone IIA on FASN, ACC1, and SCD1 protein expression in HepG2 cells. HepG2 cells were treated with vehicle (0.1% DMSO) or tanshinone IIA (5 and 10 μM) for 24 h. The protein expression of (**a**) FASN, (**b**) ACC1, and (**c**) SCD1 was measured by Western blot analysis. A representative blot is shown. The normalized intensities of (**d**) FASN, (**e**) ACC1, and (**f**) SCD1 proteins versus actin are presented as the mean ± SD of three independent experiments. # *p* < 0.05 and ## *p* < 0.01 indicate significant differences compared to the vehicle-treated cells.

Furthermore, we examined the levels of the FASN, ACC1, and SCD1 proteins in tanshinone IIA-treated HepG2 cells. The cells treated with tanshinone IIA (5 and 10 μM) had significantly decreased levels of FASN, ACC1, and SCD1 protein expression, by 0.80 ± 0.13- and 0.67 ± 0.18-fold; 0.57 ± 0.19- and 0.45 ± 0.17-fold; and 0.66 ± 0.19- and 0.43 ± 0.14-fold in HepG2 cells, respectively, when compared to the vehicle-treated cells (1.00 ± 0.03, 1.01 ± 0.08, and 1.00 ± 0.08) (*p* < 0.05 and *p* < 0.01, respectively) (Figure 3a–f). These results indicated that tanshinone IIA can significantly inhibit the mRNA and protein expression of these genes, which are essential for de novo lipogenesis in hepatic cells.

### 3.3. Effects of Tanshinone IIA on ACC1 Phosphorylation in HepG2 Cells

**Figure 4:**
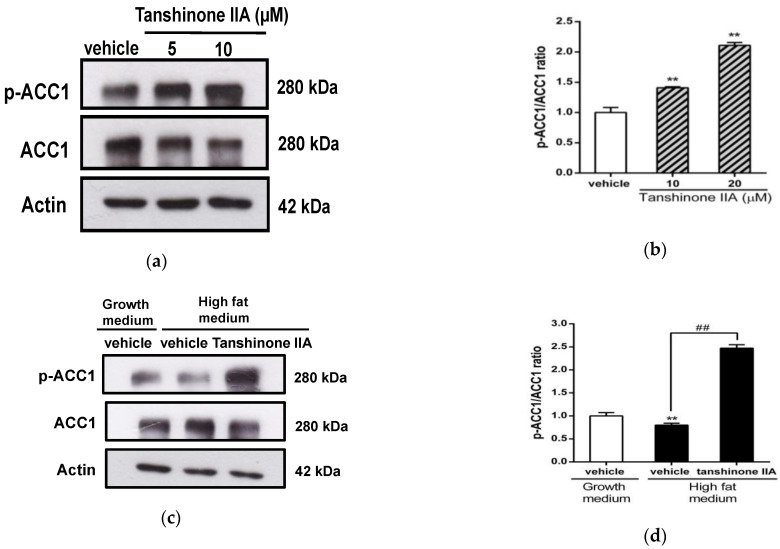
Effects of tanshinone IIA on ACC1 phosphorylation in HepG2 cells. (**a**) HepG2 cells were treated with vehicle (0.1% DMSO) or tanshinone IIA (5 and 10 μM) in normal growth medium. The protein levels of phosphorylated ACC1 at serine 79 (p-ACC1), ACC1, and actin was measured by Western blot analysis. A representative blot is shown. (**b**) The normalized intensity of p-ACC1 protein versus ACC1 is presented as the mean ± SD of three independent experiments. ** *p* < 0.01 indicates significant differences compared to the vehicle-treated cells. (**c**) HepG2 cells were treated with vehicle (0.1% DMSO) or tanshinone IIA (10 μM) in normal growth medium or high-fat medium. The phosphorylated ACC1 (p-ACC1), ACC1, and actin was measured by Western blot analysis. A representative blot is shown. (**d**) The normalized intensity of p-ACC1 versus ACC1 is presented as the mean ± SD of three independent experiments. ** *p* < 0.01 indicates significant differences compared to the vehicle-treated cells incubated with growth medium. ## *p* < 0.01 indicates significant differences compared to the vehicle-treated cells.

The enzyme ACC1 plays a critical role in fatty-acid synthesis and has been identified to be inactivated by phosphorylation of serine 79, leading to lipogenesis inhibition [45]. Therefore, we investigated the effect of tanshinone IIA on ACC1 phosphorylation in HepG2 cells. Tanshinone IIA (5 and 10 μM) significantly increased the ratio of phosphorylated ACC1 (p-ACC1)/ACC1 proteins by 1.41 ± 0.01- and 2.11 ± 0.05-fold in HepG2 cells, respectively, when compared to the vehicle-treated group (1.00 ± 0.08) (*p* < 0.01) (Figure 4a,b). Moreover, we examined the effect of tanshinone IIA on ACC1 phosphorylation in HepG2 cells incubated with high-fat medium. The ratio of p-ACC1/ACC1 in the cells treated with high-fat medium was slightly reduced by 0.80 ± 0.04-fold as compared to cells treated with normal growth medium (1.00 ± 0.07) (*p* < 0.01). Tanshinone IIA significantly increased the p-ACC1/ACC1 ratio by 2.47 ± 0.08-fold, when compared to the vehicle-treated group (0.80 ± 0.04) (*p* < 0.01) (Figure 4c,d). This data indicated that tanshinone IIA could promote phosphorylation of ACC1 protein in hepatic cells.

### 3.4. Effects of Tanshinone IIA on the Fatty-Acid-Induced Lipogenesis and TG Accumulation in HepG2 Cells

**Figure 5:**
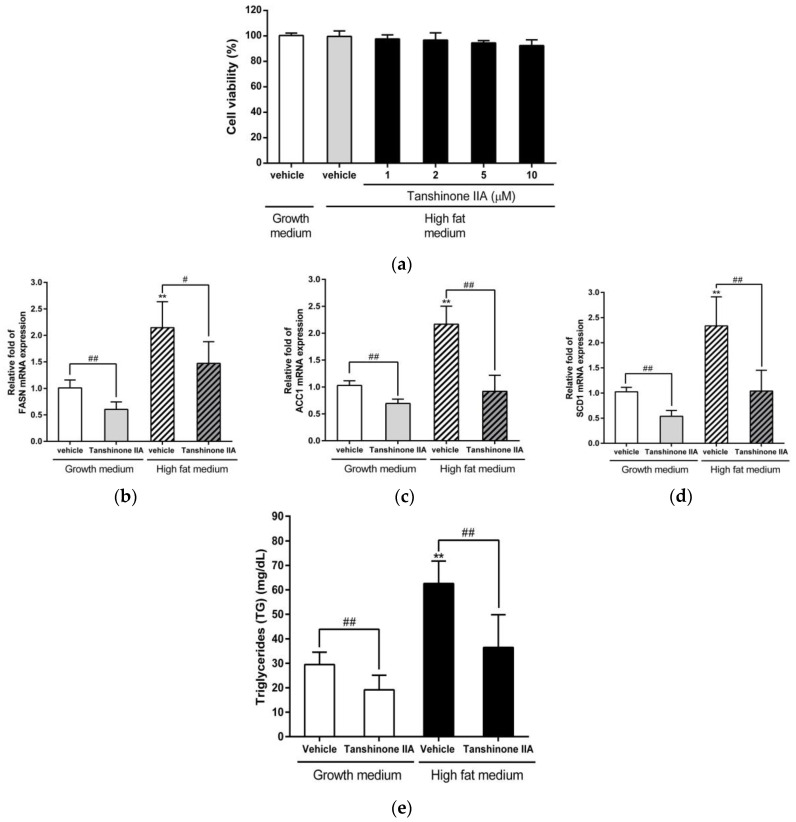
Effects of tanshinone IIA on the fatty-acid-induced lipogenesis and TG accumulation in HepG2 cells. HepG2 cells were treated with vehicle (0.1% DMSO) or tanshinone IIA (1–10 μM) in growth medium (DMEM with 10% FBS and 1xNEAA), and after 20 h, the medium was replaced with high-fat medium containing the indicated compounds (vehicle or tanshinone IIA). (**a**) Cell viability was measured using an MTT assay. The mRNA expression of (**b**) FASN, (**c**) ACC1, and (**d**) SCD1 was measured by RT-qPCR analysis. The data represent the mean ± SD of three independent experiments. # *p* < 0.05 and ## *p* < 0.01 indicate significant differences compared to the vehicle-treated cells. (**e**) Intracellular triglyceride (TG) levels of vehicle- or tanshinone-IIA-treated HepG2 cells were measured from three independent replicates, and the data represent the mean ± SD. ** *p* < 0.01 indicates significant differences compared to the vehicle-treated cells incubated with growth medium. ## *p* < 0.01 indicate significant differences compared to the tanshinone IIA-untreated cells.

To investigate the effect of tanshinone IIA on the fatty-acid-induced lipogenesis and TG accumulation, first, we examined whether tanshinone IIA affected cell viability of HepG2 cells incubated in high-fat medium. The MTT assay data showed that the viability of cells treated with tanshinone IIA (1, 2, 5, and 10 μM) was 97.7 ± 3.1%, 96.8 ± 5.6%, 94.6 ± 1.8%, and 92.4 ± 4.5% as compared to cells incubated in normal growth medium (100.4 ± 1.8%), respectively (Figure 5a). This data showed that tanshinone IIA did not induce significant cytotoxicity in cells incubated with high-fat medium. Next, the effect of tanshinone IIA on the palmitate/oleate-induced lipogenesis was investigated. The HepG2 cells incubated with high-fat medium showed increased levels of FASN, ACC1, and SCD1 mRNA expression as compared with that cultured in growth medium. Tanshinone IIA significantly decreased the amounts of FASN, ACC1, and SCD1 mRNA in cells incubated with normal growth or high-fat medium, when compared to their effects on vehicle-treated cells (*p* < 0.01) (Figure 5b–d). The effect of tanshinone IIA on intracellular triglyceride (TG) accumulation was examined, and the data showed that TG contents in cells incubated with high-fat medium were significantly increased as compared with cells treated with normal growth medium (*p* < 0.01). The TG contents were significantly reduced by tanshinone IIA (10 μM) treatment in HepG2 cells cultured in growth medium or high-fat medium (Figure 5e). These data indicated that tanshinone IIA can significantly ameliorate fatty-acid-induced lipogenesis and TG accumulation in hepatic cells.

### 3.5. Effects of Tanshinone IIA on SREBP1 Expression in HepG2 Cells

**Figure 6:**
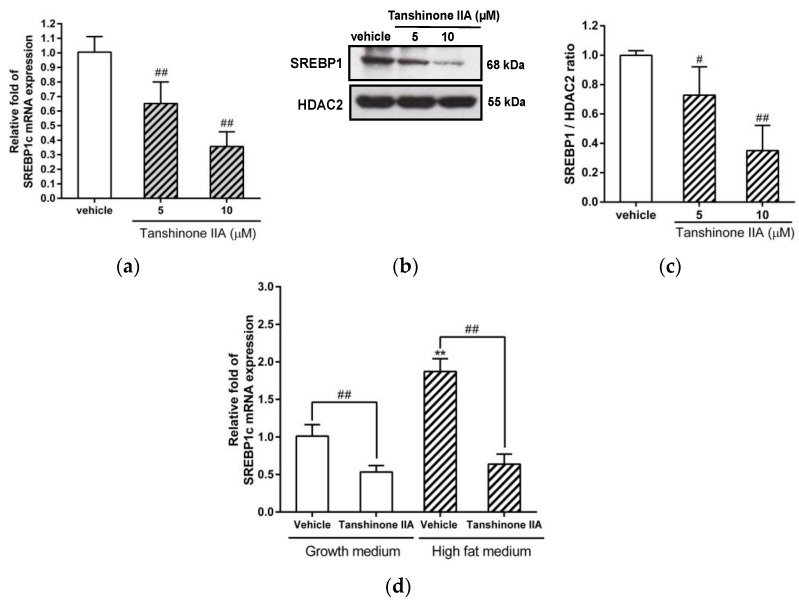
Effects of tanshinone IIA on SREBP1 gene expression. HepG2 cells were treated with vehicle (0.1% DMSO) or tanshinone IIA (5 and 10 μM) for 24 h. (**a**) The mRNA expression of SREBP-1c was measured by RT-qPCR analysis. The data represent the mean ± SD of three independent experiments. ## *p* < 0.01 indicates significant differences compared to the vehicle-treated cells. (**b**) Nuclear extracts were prepared from vehicle- or tanshinone-IIA-treated cells, and the level of nuclear SREBP1 protein was analyzed by Western blot analysis. A representative blot is shown. (**c**) The normalized intensity of SREBP1 protein versus HDAC2 is presented as the mean ± SD of three independent experiments. # *p* < 0.05 and ## *p* < 0.01 indicate significant differences compared to the vehicle-treated cells. (**d**) HepG2 cells were treated with vehicle (0.1% DMSO) or tanshinone IIA (10 μM) in normal growth medium or high-fat medium. The mRNA expression of SREBP-1c was measured by RT-qPCR analysis. The data represent the mean ± SD of three independent experiments. ** *p* < 0.01 indicates significant differences compared to the vehicle-treated cells incubated with growth medium. ## *p* < 0.01 indicates significant differences compared to the vehicle-treated cells.

SREBP-1c is an important transcriptional regulator of FASN, ACC1, and SCD1 gene expression in hepatic cells. Therefore, we further investigated the effect of tanshinone IIA on SREBP-1c expression in HepG2 cells. Tanshinone IIA (5 and 10 μM) significantly reduced the level of SREBP-1c mRNA by 0.65 ± 0.15- and 0.37 ± 0.10-fold in HepG2 cells, respectively, when compared to the vehicle-treated group (1.01 ± 0.11) (*p* < 0.01) (Figure 6a). Because the SREBP1 antibody used was unable to distinguish between the SREBP-1a and -1c, we used the SREBP1 to mention in this data.. We found that tanshinone IIA (5 and 10 μM) decreased the amount of nuclear mature SREBP1 protein by 0.73 ± 0.19- and 0.35 ± 0.17-fold, respectively, when compared to the vehicle-treated group (1.01 ± 0.03) (*p* < 0.05 and *p* < 0.01, respectively) (Figure 6b,c).

Furthermore, we investigated the effect of tanshinone IIA on SREBP1c expression in HepG2 cells incubated with high-fat medium. The cells incubated with high-fat medium showed an elevated level of SREBP1c mRNA expression by 1.87 ± 0.16-fold as compared with the cells cultured in growth medium (1.01 ± 0.15) (*p* < 0.01). Tanshinone IIA significantly decreased the amount of SREBP1c mRNA by 0.64 ± 0.13-fold in cells incubated with high-fat medium, when compared to its effect on vehicle-treated cells (*p* < 0.01) (Figure 6d). In addition to SREBP1c, recently, carbohydrate regulatory binding protein (ChREBP) also was reported to regulate the FASN and ACC1 gene expression for glucose-induced lipogenesis [2,46]. We further examined the effect of tanshinone IIA on ChREBP expression in HepG2 cells. We found that tanshinone IIA (5 and 10 μM) could not change the level of ChREBP mRNA expression (Figure S3). These above results indicated that tanshinone IIA downregulates SREBP1 gene expression, which may lead to reduce expression of lipogenic genes in hepatic cells.

### 3.6. Effects of Tanshinone IIA on LXRα-Mediated Transcriptional Activity in HepG2 Cells

**Figure 7:**
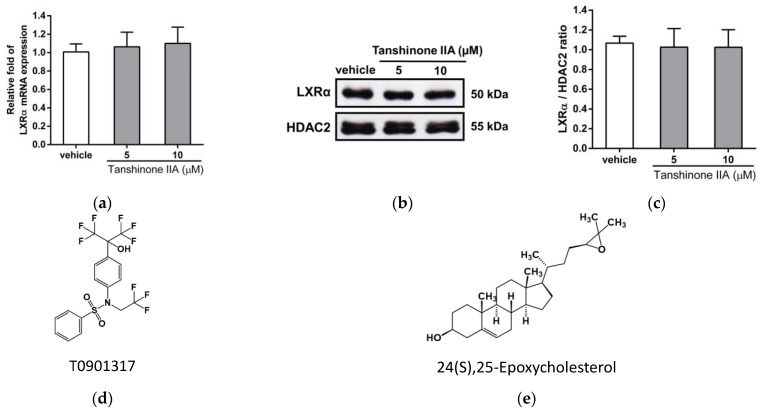

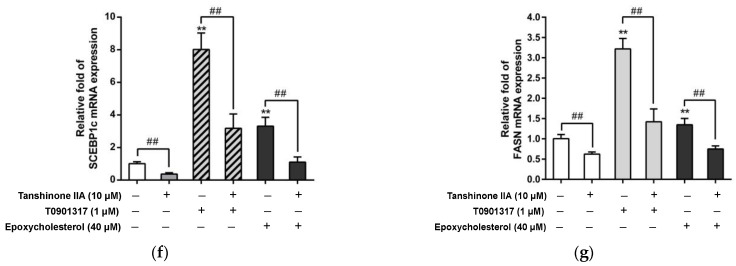
Effects of tanshinone IIA on LXRα expression and ligand-induced LXRα activation in HepG2 cells. HepG2 cells were treated with vehicle (0.1% DMSO) or tanshinone IIA (5 and 10 μM) for 24 h. (**a**) The mRNA expression of LXRα was measured by RT-qPCR analysis. The data are presented as the mean ± SD of three independent experiments. (**b**) The nuclear protein expression of LXRα and HDAC2 was measured by Western blot analysis. A represent blot is shown. (**c**) Normalized intensities of LXRα versus HDAC2 is presented as the mean ± SD of three independent experiments. The chemical structure of (**d**) T0901317 and (**e**) 24(S),25-epoxycholesterol HepG2 cells were pretreated with vehicle (0.1% DMSO) or tanshinone IIA (10 μM) for 1 h followed by treatment with agonist T0901317 (1 μM) or 24(S),25-epoxycholesterol (40 μM) for 24 h. The mRNA levels of (**f**) SREBP-1c and (**g**) FASN were measured by RT-qPCR analysis. ** *p* < 0.01 indicates a significant difference compared to compound-untreated group. ## *p* < 0.01 indicates significant differences compared to cells treated with the agonist alone.

SREBP-1c and some lipogenic enzymes such as FASN are the direct targets for transcriptional activation by LXRα in hepatic cells. To explore the effect of tanshinone IIA on LXRα-mediated SREBP1 expression, we first examined whether tanshinone IIA could change LXRα gene expression in HepG2 cells. We found that tanshinone IIA did not change the mRNA or nuclear protein levels of LXRα in the HepG2 cells (Figure 7a–c). We further examined the effect of tanshinone IIA on the ligand-induced LXR activity for SREBP-1c mRNA expression. HepG2 cells were pretreated with vehicle or tanshinone IIA (10 μM) for 1 h followed by treatment with LXRα agonists; namely, T0901317 (Figure 7d) and 24(S),25-epoxycholesterol (Figure 7e), for an additional 24 h. As shown in Figure 7f, the level of SREBP-1c mRNA was significantly increased by T0901317 (1 μM) and 24(S),25-epoxycholesterol (40 μM) treatment of the HepG2 cells. When cells were cotreated with tanshinone IIA and agonists, the T0901317- and 24(S),25-epoxycholesterol-induced SREBP-1c mRNA expression was significantly attenuated by approximately 60% and 67%, respectively (*p* < 0.01). Furthermore, we found that T0901317- and 24(S),25-epoxycholesterol-induced FASN mRNA expression was also significantly decreased by approximately 56% and 45%, respectively, in tanshinone IIA-treated cells (*p* < 0.01) (Figure 7g). Moreover, to validate the regulatory effect of tanshinone IIA on LXRα-mediated transcription, mRNA expression of other downstream target genes, such as ACC1 and SCD1 [24,47], and GLUT1 [48], a negatively regulated gene by LXRα ligands, were also examined. T0901317-induced ACC1 and SCD1 mRNA expression was significantly decreased by tanshinone IIA in HepG2 cells (Figure S4a,b). Tanshinone IIA also could reverse the T0901317-decreased GLUT1 expression to basal level in HepG2 cells (Figure S4c). These above results verified that tanshinone IIA was involved in the regulation of LXRα-mediated transcriptional activity in hepatic cells.

### 3.7. Tanshinone IIA Attenuates LXRα-Mediated Lipid-Droplet Accumulation in HepG2 Cells

**Figure 8:**
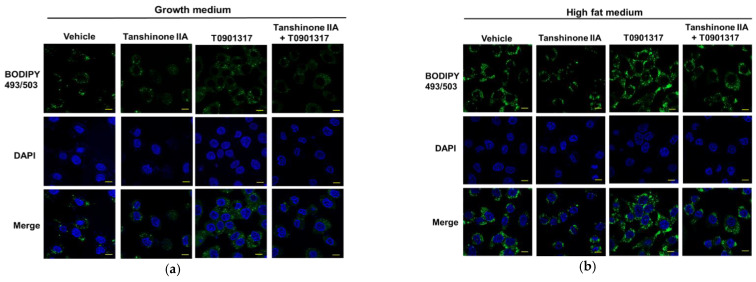

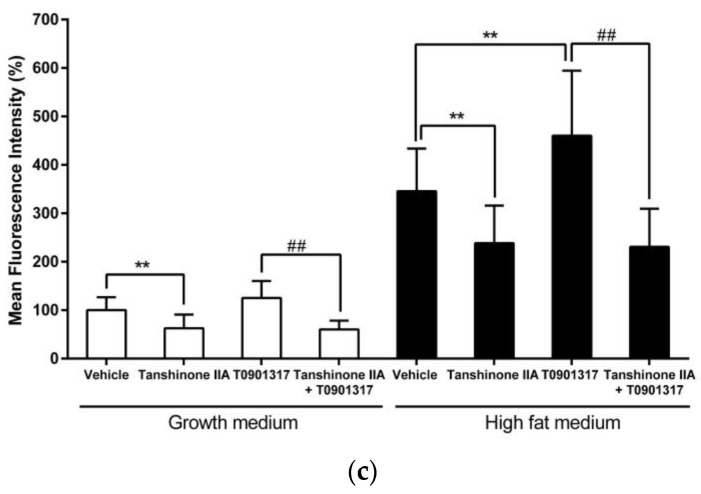
Effects of tanshinone IIA on lipid-droplet accumulation in HepG2 cells. HepG2 cells were pretreated with vehicle (0.1% DMSO) or tanshinone IIA (10 μM) for 1 h followed by treatment with T0901317 (1 μM) in (**a**) growth medium or (**b**) high-fat medium. The lipid droplets were stained by BODIPY493/503 fluorescent dye (green), and nuclei were stained by DAPI (blue). Stained cells were observed and photographed with a confocal microscope. The scale bar is 10 μm. (**c**) Quantification of lipid droplets accumulation. The mean fluorescent intensities of the lipid droplets per cell were quantified in 25–30 randomly selected fields (>200 cells) from three independent replicates, and the data represent the mean ± SD from three independent experiments. ** *p* < 0.01 indicates significant differences compared to the vehicle-treated cells. ## *p* < 0.01 indicates significant differences compared to the cells treated with T0901317 alone.

To investigate the effect of tanshinone IIA on lipid accumulation in HepG2 cells, the neutral lipid droplets in HepG2 cells were assessed using BODIPY493/503 fluorescent dye staining and analyzed under a confocal microscope. The cells incubated with high-fat medium showed an increased level of lipid droplets as compared with the cells cultured in normal growth medium (Figure 8a,b). Tanshinone IIA could significantly reduce the amount of lipid droplets in cells incubated with growth medium and high-fat medium by approximately 37% and 32%, respectively, when compared to its effect on vehicle-treated cells (Figure 8c). We further investigated the effect of tanshinone IIA on LXRα-mediated lipid-droplet accumulation in HepG2 cells. The data from Figure 8a–c showed that lipid accumulation induced by the T0901317 (1 μM) in cells with growth or high-fat medium was increased by approximately 25% and 33%, respectively, when compared to vehicle-treated groups. When cells were cotreated with T0901317 and tanshinone IIA (10 μM), the T0901317-induced lipid droplets were significantly attenuated by approximately 52% and 49% in cells incubated with growth medium and high-fat medium, respectively. These data indicated that tanshinone IIA can attenuate LXRα-mediated lipid droplets accumulation in HepG2 cells.

### 3.8. Tanshinone IIA Docks to the Ligand-Binding Domain of LXRα

**Figure 9:**
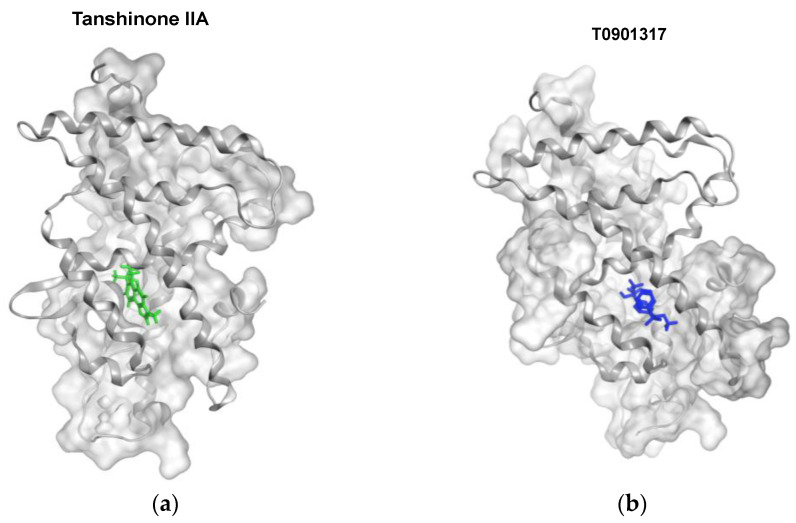

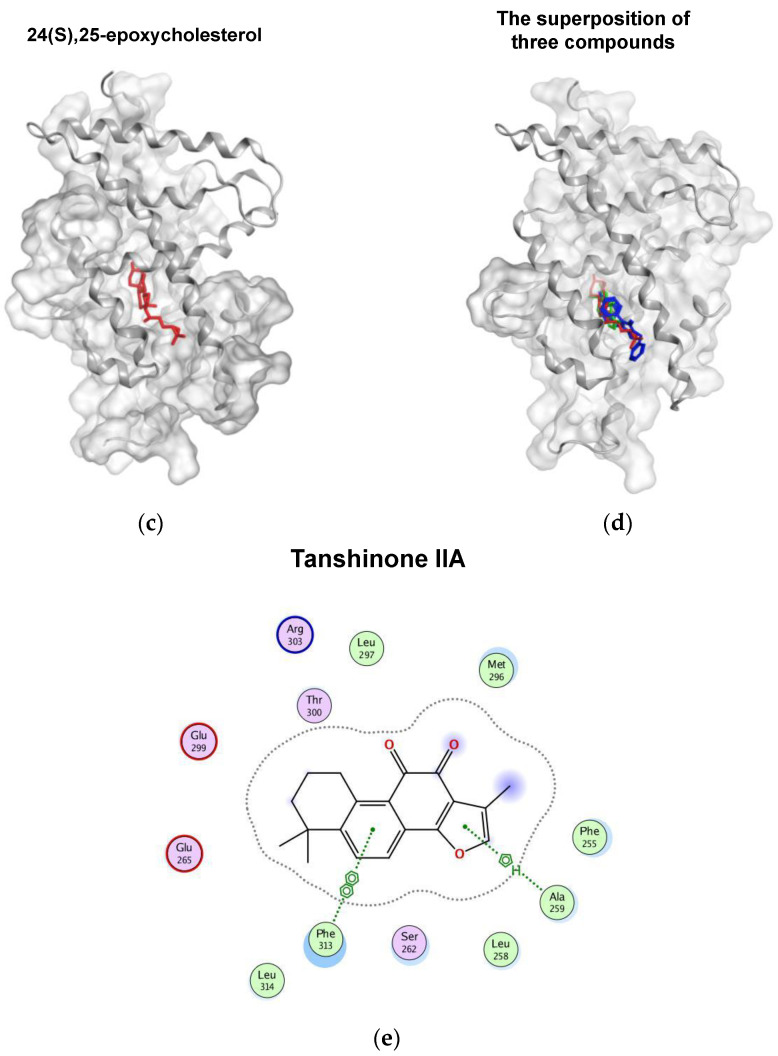
Tanshinone IIA docks to the ligand-binding domain of LXRα. The poses of compounds docked preferentially to the ligand-binding domain of LXRα. The receptor is shown as a gray ribbon, and the compounds are shown in (**a**) green (tanshinone IIA), (**b**) blue (T0901317), and (**c**) red (24(S),25-epoxycholesterol). (**d**) The superposition of these three compounds bound to the ligand-binding domain pocket of LXRα. Two-dimensional interaction map of LXRα with (**e**) tanshinone IIA. Many hydrophobic amino- acid residues (green color) surrounded the tanshinone IIA.

To investigate whether tanshinone IIA can bind to LXRα, the molecular docking program was performed as described in the Materials and Methods section. The docking results showed that tanshinone IIA, T0901317, and 24(S),25-epoxycholesterol can dock to LXRα binding pocket with different docking scores (tanshinone IIA: −7.33; T0901317: −8.47; and 24(S),25-epoxycholesterol: −10.32) (Figure 9a–c). The superposition result showed that all these compounds docked into the ligand-binding domain of LXRα at similar poses (Figure 9d)**.** The ligand-receptor interaction maps showed that the tanshinone IIA was surrounded by many hydrophobic amino-acid residues such as F255, L258, A259, M296, and F313 (Figure 9e). These data indicated that the binding pocket of LXRα is suitable for lipophilic molecule interactions and tanshinone IIA may serve as a ligand specifically bound to this ligand-binding domain for modulation of LXRα activity.

## 4. Discussion

**Figure 10:**
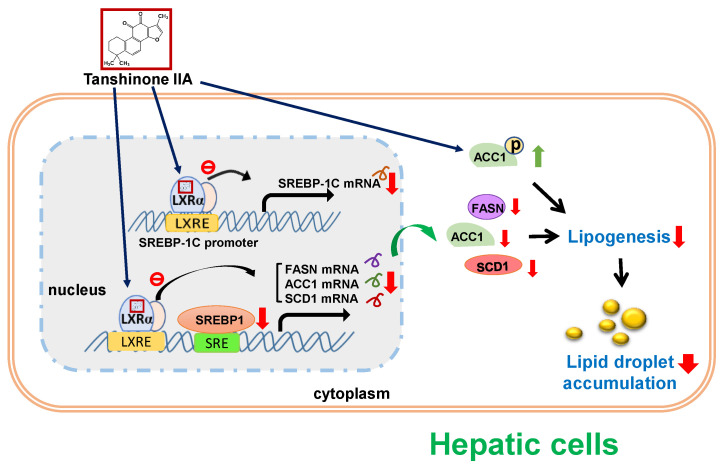
A hypothetical model for the tanshinone-IIA-mediated attenuation of lipid accumulation in hepatic cells. Tanshinone IIA promotes ACC1 phosphorylation to attenuate fatty-acid synthesis and lipogenesis. Tanshinone IIA down-regulates FASN, ACC1, and SCD1 expression by inhibiting the LXRα-mediated transcriptional activation, resulting in a reduction in endogenous and fatty-acid-induced lipogenesis as well as lipid-droplet accumulation in hepatic cells.

Abnormal accumulation of lipid droplets within hepatic cells is the hallmark of MAFLD. MAFLD patients may benefit from early treatment to reduce hepatic or cardiovascular complications [49]. Tanshinone IIA, a pharmacologically bioactive phytochemical extracted from the dried root of *Salvia miltiorrhiza*, has been demonstrated to have putative effects on antiatherosclerosis, antihyperlipidemia and antiadipogenesis [50]. Recently, tanshinone IIA and its derived compound, sodium tanshinone IIA sulfonate, were reported to ameliorate hepatic steatosis or fatty liver [35,51,52]. However, studies of the molecular effects of tanshinone IIA on hepatic steatosis and its role in the prevention or treatment of MAFLD remain limited. In this study, we demonstrated that tanshinone IIA could significantly reduce the amounts of lipid-droplet deposition within HepG2 cells. Tanshinone IIA could increase the phosphorylation of ACC1 proteins, which may lead to inhibit hepatic lipogenesis. Tanshinone IIA downregulated the expression of lipogenic enzymes, including FASN, ACC1, and SCD1, through suppression of SREBP1 expression and LXRα-mediated transcriptional activation, resulting in reducing lipogenesis and lipid accumulation in the HepG2 cells. Moreover, we predicted that tanshinone IIA binds to the ligand-binding domain of LXRα to suppress its transcriptional activity (Figure 10). These findings support the hypothesis that tanshinone IIA possesses lipid-modulating activity and can potentially serve as a novel agent for the prevention or treatment of hepatic steatosis or MAFLD.

Hepatic steatosis results from an imbalance between lipid supplementation and clearance in the liver. Emerging studies have demonstrated that dysregulation of lipogenesis contributes to hepatic steatosis and plays an essential role in the pathologic progress of MAFLD. The increases in hepatic TG content measured in MAFLD patients showed that approximately 30% of the contribution came from de novo biosynthesis [17]. In this study, tanshinone IIA downregulated the mRNA and protein expression of lipogenic enzymes, including FASN, ACC1, and SCD1, in HepG2 cells. Our data also showed tanshinone IIA markedly reduced TG contents and lipid-droplet accumulation. It has been reported that modulation of de novo lipogenesis in hepatocytes is important for the attenuation of hepatic steatosis and treatment of MAFLD. Recent studies using animal models have shown that knocking out or knocking down the hepatic enzymes involved in lipogenesis or desaturation of long-chain fatty acids may reduce TG concentration and attenuate steatosis [16,53,54]. The present study revealed that tanshinone IIA can reduce the levels of lipogenic enzymes in hepatocytes, which may lead to decreased hepatic TG contents and lipid accumulation. Saturated-fat diets could increase lipogenic gene expression and hepatic steatosis in mice [55]. The TG-derived fatty-acid profile showed that higher saturated fatty-acid content and low levels of polyunsaturated fatty acid increased the proinflammatory cytokine to enhance inflammation [56]. In this study, inhibition of palmitate/oleate-induced lipogenesis by tanshinone IIA may be associated with reduction of TG accumulation. Whether tanshinone IIA could increase the unsaturated fatty-acid levels and reduce saturated fatty-acid content in cells treated with high-fat medium remains unclear. Analysis of the profile of TG-derived saturated and mono/polyunsaturated fatty acid in lipid droplets from tanshinone IIA-treated cells needs to be further investigated.

In this study, tanshinone IIA increased the ratio of p-ACC1/ACC1 in HepG2 cells. These data indicated that tanshinone IIA could promote ACC1 phosphorylation, which may lead to inhibit its enzyme activity for the decrease of de novo lipid biosynthesis. It is known that AMPK activation directly phosphorylated ACC1 at serine 79 and suppressed its activity, and reduced FASN expression to attenuate fatty-acid-induced hepatic steatosis. AMPK activation also decreased fatty-acid biosynthesis through reducing the SREBP1c levels or nuclear translocation to repress lipogenic gene expression and lipid accumulation [57,58]. Recently, AMPK activator was reported to modulate hepatic saturated/unsaturated fatty-acid composition, and suppress hepatic macrophage infiltration in the development of MAFLD [59]. Whether tanshinone IIA can activate AMPK signaling to regulate lipogenic gene expression and reduce hepatic steatosis remains unclear and needs to be further investigated.

Transcriptional modulation of the lipogenic enzymes in hepatocytes is an interesting idea for developing therapeutic strategies for hepatic steatosis. The transcription factor SREBP1 regulates the transcription rate of genes involved in the fatty-acid synthesis pathway. SREBP1 is expressed in two isoforms, SREBP-1a and SREBP-1c, can be translated as a precursor protein bound to the endoplasmic reticulum (ER) membrane, and must be transported to the Golgi and activated by a cleavage process to release a mature form to the nucleus [60]. SREBP1 mediates the regulation of hepatic genes involved in carbohydrate and lipid metabolism. SREBP-1c was reported to control the mRNA expression of FASN, ACC1, and SCD1 in hepatocytes [61,62]. Additionally, hepatic SREBP-1c was highly expressed in patients with MAFLD [63]. In this study, we found that tanshinone IIA significantly decreased the levels of SREBP-1c mRNA and the mature form of nuclear SREBP1 protein. These results revealed that tanshinone IIA can inhibit the transcription of FASN, ACC1, and SCD1 via the modulation of nuclear SREBP1 levels in hepatic cells.

The essential role of SREBP1 in lipogenesis has been demonstrated in gain- and loss-of-function studies in vitro and in vivo. Overexpressing a mature form of SREBP-1c in hepatocytes or mice can activate the lipogenic pathway and induce steatosis [64,65]. In contrast, mice lacking SREBP-1c fail to express of lipogenic enzymes or undergo TG synthesis in response to fasting and refeeding [23]. The data we presented here demonstrated that tanshinone IIA could cause a significant decrease in the level of the mature SREBP1 protein. These findings indicate that tanshinone-IIA-mediated nuclear SREBP1 reduction leads to the downregulated expression of lipogenic enzymes and attenuates lipid accumulation in hepatic cells. SREBP1 protein is also known to play a critical role in hepatic carbohydrate metabolism [66]. Whether tanshinone-IIA-mediated SREBP1 downregulation modulates glucose metabolism in hepatic cells needs to be further investigated.

The SREBP-1c gene is upregulated by the nuclear receptor LXRα. LXR is expressed in two isoforms, LXRα and LXRβ, and LXRs are ligand-dependent nuclear receptors that regulate cholesterol and lipid metabolism [67,68]. LXRα is mainly expressed in the liver, kidney, adipose tissue, and intestine, whereas LXRβ is ubiquitously expressed [69]. LXRα agonist treatment has been reported to induce lipogenesis, because they bind to LXREs within the promoter region of SREBP-1c, FASN, ACC1, and SCD1 to increase mRNA transcription in hepatic cells [24,47,70,71]. LXRα and its lipogenic target genes were also reported to be highly expressed in patients with MAFLD [72]. In this study, we found that the LXR agonist enhanced lipogenic genes and contributed to lipid accumulation as expected in HepG2 cells. In tanshinone-IIA-treated cells, LXRα-induced lipid-droplet accumulation can be significantly attenuated. Lipid droplets are dynamic lipid-rich organelles that have been shown to function in TG and energy storage, and as a protective strategy for fatty-acid-induced lipotoxicity in the liver. However, an imbalance of lipid-droplet formation and mobilization can lead to excess lipid droplets and TG accumulation in hepatic cells, which is a pathological condition for MAFLD [73,74]. In this study, tanshinone IIA significantly ameliorated both fatty-acid- and LXRα-induced lipid-droplet accumulation in HepG2 cells. This finding suggested that tanshinone IIA may modulate lipid-droplet formation and homeostasis in the liver.

The LXRα protein is composed of an N-terminal-activating domain, a DNA-binding domain, a ligand-binding domain (LBD), and a C-terminal activation domain. The LBD of LXRα has been reported to form a hydrophobic pocket and may interact with small-molecule compounds. The binding affinity of LXR to its ligand could be predicted by molecular-docking analysis [75]. In this study, molecular docking predicted that tanshinone IIA specifically binds to the LBD of LXRα. The lipophilic tanshinone IIA seems preferentially to interact with hydrophobic residues within the LBD pocket of LXRα. Thus, we suggested that the LXRα-ligand-induced lipid accumulation could be significantly ameliorated by tanshinone IIA. LXR agonists were reported to increase expression of their downstream target genes such as ACC1 and SCD1, and decrease GLUT1 expression. In this study, tanshinone IIA could counteract the effect of the LXRα ligand T0901317 on these target genes’ expression; however, LXRα mRNA and protein levels were not changed. These results supported that tanshinone IIA could serve as a novel LXRα ligand to modulate the LXRα–SREBP1 axis in hepatic cells. The precise mechanisms underlying the suppression of LXRα activation by tanshinone IIA remain unclear. The determination of tanshinone IIA as an LXR antagonist, an inhibitor, or a ligand competitor that blocks the LXR-RXR heterodimer needs further functional studies, with subjects such as GLUT1 or other downstream targets involved in glucose or lipid metabolism.

LXRα plays essential roles in fatty-acid and cholesterol homeostasis, and in control of inflammation and immunity [76,77]. LXR activation may control cholesterol efflux from foam cells, induce cholesterol transporter expression, and inhibit inflammatory mediators in the artery wall and macrophage, which may lead to reduced vascular inflammation and atherosclerosis [49]. Therefore, LXR agonists are an option to be used for the treatment of atherosclerosis, diabetes, and other disorders [78]. However, LXR agonists activated hepatic lipogenesis and promoted TG accumulation in MAFLD, which limit the value of clinical application. In this study, tanshinone IIA may have served as a ligand and counteracted the LXRα–SREBP1 axis to reduce hepatic lipid accumulation. Recently, tanshinone IIA treatment was reported to reduced macrophage infiltration and nuclear factor (NF)-κB activation in macrophages for inhibiting inflammatory responses [79]. Tan et al. reported that tanshinone IIA increased cholesterol efflux and reduced lipid accumulation in macrophages, leading to a reduction in the development of aortic atherosclerosis [80]. Wen et al. reported that tanshinone IIA inhibited oxidized LDL-induced NLRP3 inflammasome activation in mouse macrophages and ameliorated atherogenesis [81]. These findings indicated that tanshinone IIA possessed the potential activities for anti-inflammation and antiatherosclerosis. In this study, we suggested that tanshinone IIA could attenuate ligand-induced LXRα activation and may be an LXRα antagonist or a ligand competitor for downregulation of lipogenic gene expression in hepatic cells. Whether tanshinone IIA affects the anti-inflammatory effect and cholesterol accumulation by LXRα-dependent pathways in extra-hepatocytes remains to be clarified, and needs to be considered with caution due to its adverse effect in therapeutic strategy for treatment of MAFLD. Recently, Griffett et al. reported that a hepato-selective LXR inverse agonist could specifically suppress LXR activity in hepatic cells to reduce lipogenesis and prevent MAFLD [82]. Whether tanshinone IIA could serve as an inverse agonist to reduce lipogenesis without promoting cholesterol accumulation in extra-hepatic tissue remains to be further investigated. In this study, we realized our results were found only on the in vitro lipid-lowering effects of tanshinone IIA on hepatic cancer cell lines. To verify the impact of treatment with tanshinone IIA in vivo, the primary human hepatocytes and animal models of MAFLD can be used in further studies of tanshinone IIA on modulation of lipogenesis and lipid accumulation.

## 5. Conclusions

In this study, for the first time, we demonstrated that tanshinone IIA exerts a promising lipid-modulating effect to attenuate lipid-droplet accumulation and downregulate the expression of genes involved in de novo lipogenesis by regulating the LXRα/SREBP1 pathway in hepatic cells. Our findings revealed that tanshinone IIA may serve as a novel supplement or drug to regulate LXRα activity and counter unwanted lipid overproduction in the liver for the prevention or treatment of MAFLD.

## Data Availability

Not applicable.

## Supplementary Materials

The following are available online at https://www.mdpi.com/2227-9059/9/3/326/s1, Table S1: The primer pairs used in RT-qPCR, Figure S1: Effects of tanshinone IIA on Huh 7 cell viability, Figure S2: Effects of tanshinone IIA on FASN, ACC1, and SCD1 mRNA expression in Huh 7 cells, Figure S3: Effects of tanshinone IIA on ChREBP mRNA expression in HepG2 cells, Figure S4: Effects of tanshinone IIA on LXRα-mediated transcriptional activity in HepG2 cells.
