# Supplementary material for: Tanshinone IIA Downregulates Lipogenic Gene Expression and Attenuates Lipid Accumulation through the Modulation of LXRα/SREBP1 Pathway in HepG2 Cells
Source: Biomedicines. 2021 Mar 23;9(3):326. doi: 10.3390/biomedicines9030326 (PMC8004631; doi:10.3390/biomedicines9030326)
Supplement: Supplementary file 1 [file biomedicines-09-00326-s001.pdf]

biomedicines

Supplementary Materials

Table S1. The primer pairs used in RT-qPCR.

Genes	Primers
FASN	5′-CCCCTGATGAAGAAGGATCA-3′
	5′-ACTCCACAGGTGGGAACAAG-3′
ACC1	5′-AGTGAGGATGGCAGCTCTGGA-3′
	5′-TGAGATGTGGGCAGCATGAAC-3′
SCD1	5′-GCAGGACGATATCTCTAGCT-3′
	5′-GTCTCCAACTTATCTCCTCCATTC-3′
SREBP-1c	5′-GGAGGGGTAGGGCCAACGGCCT-3′
	5′-CATGTCTTCGAAAGTGCAATCC-3′
LXRα	5′-GCCGAGTTTGCCTTGCTCA-3′
	5′-TCCGGAGGCTCACCAGTTTC-3′
ChREBP	5′-CAGCTGCGGGATGAGATTGA-3′
	5′-AAACGCTGGTGTGTGATGGGTA-3′
GLUT1	5′-CGGGCCAAGAGTGTGCTAAA-3′
	5′-TGACGATACCGGAGCCAATG-3′
GAPDH	5′-ATGAGAAGTATGACAACAGCCT-3′
	5′-AGTCCTTCCACGATACCAAAGT-3′

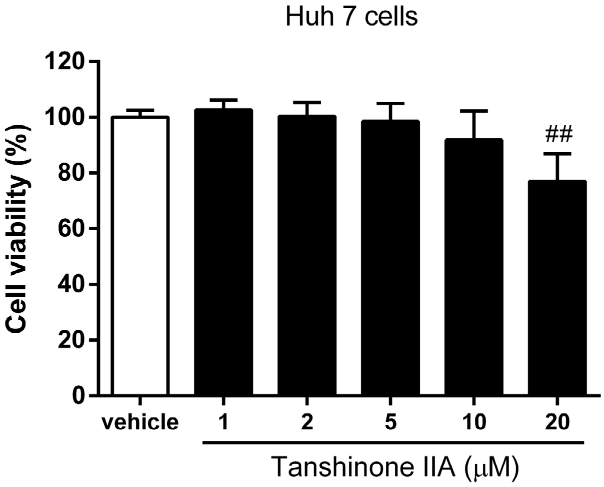

Figure S1. Effects of tanshinone IIA on Huh 7 cell viability. Huh 7 cells were treated with vehicle (0.1% DMSO) or tanshinone IIA (1,2,5,10 and 20 μM) for 24 h. Cell viability was measured using an MTT assay. The data represent the mean ± SD of three independent experiments. ##$p < 0.01$ indicates significant differences compared to the vehicle-treated cells.

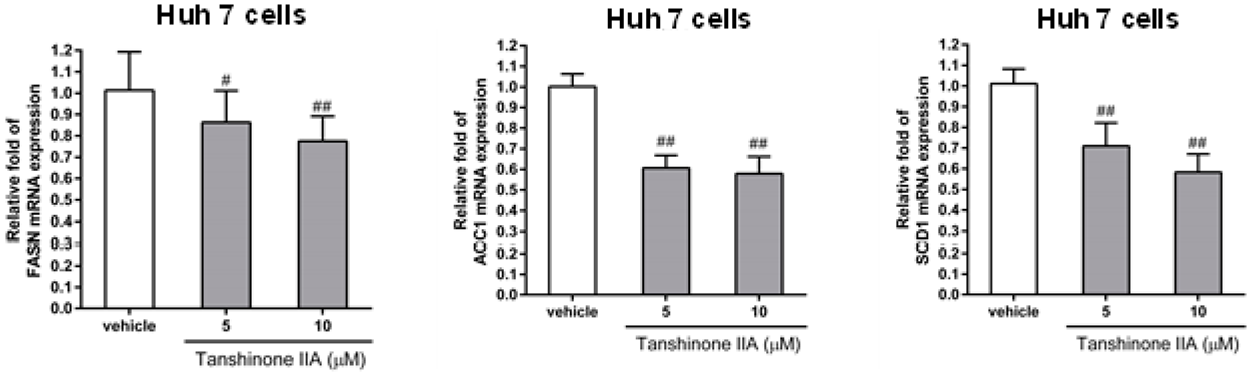

(a) (b) (c)

Figure S2. Effects of tanshinone IIA on FASN, ACC1 and SCD1 mRNA expression in Huh 7 cells. Huh 7 cells were treated with vehicle (0.1% DMSO) or tanshinone IIA (5 and 10 μM) for 24 h. The mRNA expression of (**a**) FASN, (**b**) ACC1, and (**c**) SCD1 was measured by RT-qPCR analysis. The data represent the mean ± SD of three independent experiments. #p < 0.05 and ##p < 0.01 indicate significant differences compared to the vehicle-treated cells.

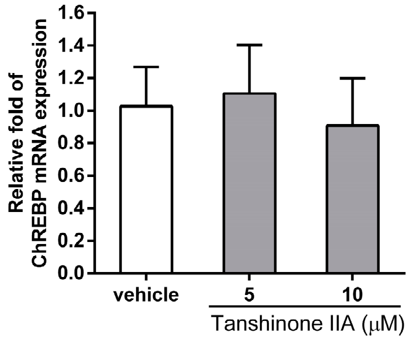

Figure S3. Effects of tanshinone IIA on ChREBP mRNA expression in HepG2 cells. HepG2 cells were treated with vehicle (0.1% DMSO) or tanshinone IIA (5 and 10 μM) for 24 h. The mRNA expression of ChREBP was measured by RT-qPCR analysis. The data represent the mean ± SD of three independent experiments.

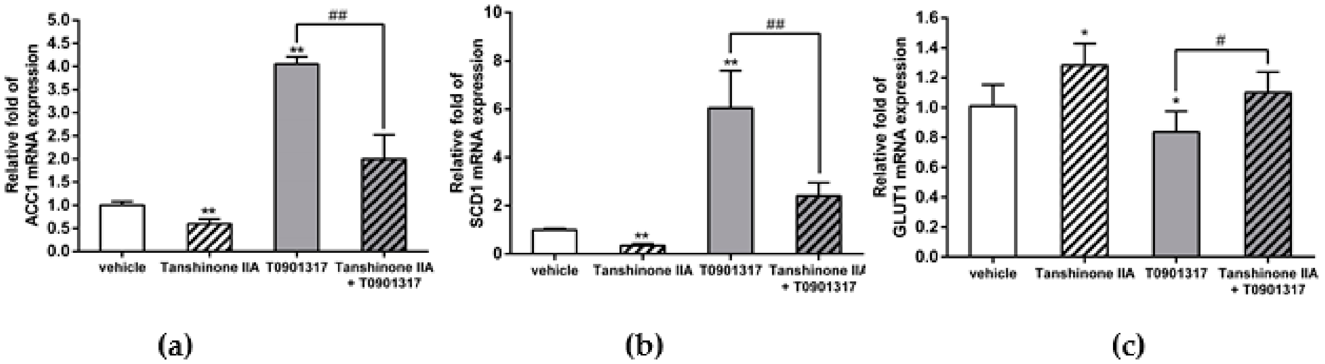

(a) (b) (c)

Figure S4. Effects of tanshinone IIA on LXRα-mediated transcriptional activity in HepG2 cells. HepG2 cells were pre-treated with vehicle (0.1% DMSO) or tanshinone IIA (10 μM) for 1 h followed by treatment with agonist T0901317 (1 μM) for 24 h. The mRNA levels of (**a**) ACC1, (**b**) SCD1 and (**c**) GLUT1 were measured by RT-qPCR analysis. *p < 0.05 and **p < 0.01 indicate a significant difference compared to vehicle-treated group. #p < 0.05 and ##p < 0.01 indicate significant differences compared to the T0901317 alone-treated cells.
